# Particulate matter (PM_2.5_) as a potential SARS-CoV-2 carrier

**DOI:** 10.1038/s41598-021-81935-9

**Published:** 2021-01-28

**Authors:** Norefrina Shafinaz Md Nor, Chee Wai Yip, Nazlina Ibrahim, Mohd Hasni Jaafar, Zetti Zainol Rashid, Norlaila Mustafa, Haris Hafizal Abd Hamid, Kuhan Chandru, Mohd Talib Latif, Phei Er Saw, Chin Yik Lin, Kemal Maulana Alhasa, Jamal Hisham Hashim, Mohd Shahrul Mohd Nadzir

**Affiliations:** 1 Department of Biological Sciences and Biotechnology, Faculty of Science and Technology, Universiti Kebangsaan Malaysia UKM, 43600 Bangi, Selangor Malaysia; 2 Department of Community Health, Faculty of Medicine, Universiti Kebangsaan Medical Centre, 56000 Kuala Lumpur, Malaysia; 3 Department of Medical Microbiology and Immunology, Faculty of Medicine, Universiti Kebangsaan Medical Centre, 56000 Kuala Lumpur, Malaysia; 4 Consultant Physician and Endocrinologist, Faculty of Medicine, Universiti Kebangsaan Medical Centre, 56000 Kuala Lumpur, Malaysia; 5 Department of Earth Sciences and Environment, Faculty of Science and Technology, Universiti Kebangsaan Malaysia, 43600 Bangi, Selangor Malaysia; 6 Guangdong Provincial Key Laboratory of Malignant Tumor Epigenetics and Gene Regulation, Sun Yat-Sen Memorial Hospital, Sun Yat-Sen University, Guangzhou, 510120 People’s Republic of China; 7 Institute of Climate Change, Space Science Centre (ANGKASA), Universiti Kebangsaan Malaysia, 43600 Bangi, Selangor Malaysia; 8 Department of Geology, Faculty of Science, University of Malaya, 50603 Kuala Lumpur, Federal Territory of Kuala Lumpur Malaysia; 9 Department of Environmental Health, Faculty of Health Sciences, Universiti Selangor, 40000 Shah Alam, Malaysia

**Keywords:** Microbiology, Environmental sciences, Diseases

## Abstract

The rapid spread of the SARS-CoV-2 in the COVID-19 pandemic had raised questions on the route of transmission of this disease. Initial understanding was that transmission originated from respiratory droplets from an infected host to a susceptible host. However, indirect contact transmission of viable virus by fomites and through aerosols has also been suggested. Herein, we report the involvement of fine indoor air particulates with a diameter of ≤ 2.5 µm (PM_2.5_) as the virus’s transport agent. PM_2.5_ was collected over four weeks during 48-h measurement intervals in four separate hospital wards containing different infected clusters in a teaching hospital in Kuala Lumpur, Malaysia. Our results indicated the highest SARS-CoV-2 RNA on PM_2.5_ in the ward with number of occupants. We suggest a link between the virus-laden PM_2.5_ and the ward’s design. Patients’ symptoms and numbers influence the number of airborne SARS-CoV-2 RNA with PM_2.5_ in an enclosed environment.

## Introduction

The Severe Acute Respiratory Syndrome Coronavirus 2 (SARS-CoV-2) is primarily transmitted via respiratory droplets of various sizes^1–3^. Large respiratory droplets (> 5 μm) transmission occur when a person is in close contact with someone^4^ who has respiratory symptoms such as coughing or sneezing^5^. Whereas, finer virus-laden respiratory droplets and particulate matters (≤ 5 μm) can remain in the air for an extended period and be carried over greater distances^6^ > 6 m (such as the outbreak of tuberculosis, measles, and chickenpox)^7^. Despite numerous studies that have demonstrated the transmission route of SARS-CoV-2 via respiratory droplets, evidence on aerosols-borne transmission remains limited^1,8,9^.

Transmission of SARS-CoV-2 in a range of particulate matter (PM) from submicrometer and/or supermicrometer have been reported^1,10^. This suggests that the virus can be transported via solid aerosols. PM_2.5_ is fine solids with a particle diameter of ≤ 2.5 μm that is suspended in ambient air aerosols.

No correlation was found between the virus concentration and PM’s diameter. Nevertheless, positive correlations between PM_2.5_ and other respiratory viruses such as the influenza virus have been reported^11^, emphasizing the possibility of particulate matter as a transport carrier for SARS-CoV-2.

PM_2.5_ is fine solid aerosols with a particle diameter of ≤ 2.5 μm that is suspended in ambient air. PM_2.5_ in indoor environments is mainly derived from common outdoor sources such as motor-vehicles, biomass burning, and industrial emissions^12–14^. Prolonged exposure to PM_2.5_ is particularly detrimental to human health as this fine particulate matter can be easily inhaled and penetrate deep into the lungs^15,16^. PM_2.5_ is known to have a significantly longer lifetime in the air where it can be suspended at an extended period compared to respiratory liquid droplets. This longer lifetime of particles may pose a significant viral exposure to healthcare personnel, especially in indoor environments. PM_2.5_ can also be deposited in indoor environments such as hospitals’ flooring^17,18^ and any surface materials^19,20^. This fine particulate matter is readily propagated by tiny turbulent eddies in the air that arise from physical activities such as human movements and walking^21,22^. Considering the fact that the viability of SARS-CoV-2 on many types of surfaces have been reported (e.g., on metals for 48 h, plastic for 72 h, cardboard for 24 h, and copper for 4 h)^23,24^, it is likely that the virus on the surface can be potentially lodged on the PM_2.5_ and redistributed/transported back into the air.

Recent findings based on air particle measurements have suggested that SARS-CoV-2 can be carried by PM_2.5_ in the air when healthcare workers remove their personal protective equipment (PPE)^2,5^. Furthermore, it is also suggested that suspended tiny dust in the air could couple with microorganisms of diameter < 5 μm during aerosolization^7^. Since the diameter of the SARS-CoV-2 is two orders of magnitude smaller—approximately 70–90 nm^25^, the mechanism/mode of the airborne transport is still unclear and, therefore, worth exploring. In this study, we hypothesize the possible role of PM_2.5_ as a carrier (or transport agent) for SARS-CoV-2 to remain in the air. In order to prove this hypothesis, we investigated the PM_2.5_ burden and SARS-CoV-2 from several wards with COVID-19 patients in a hospital.

## Methods

### Sampling location and indoor air sampling

**Figure 1:**
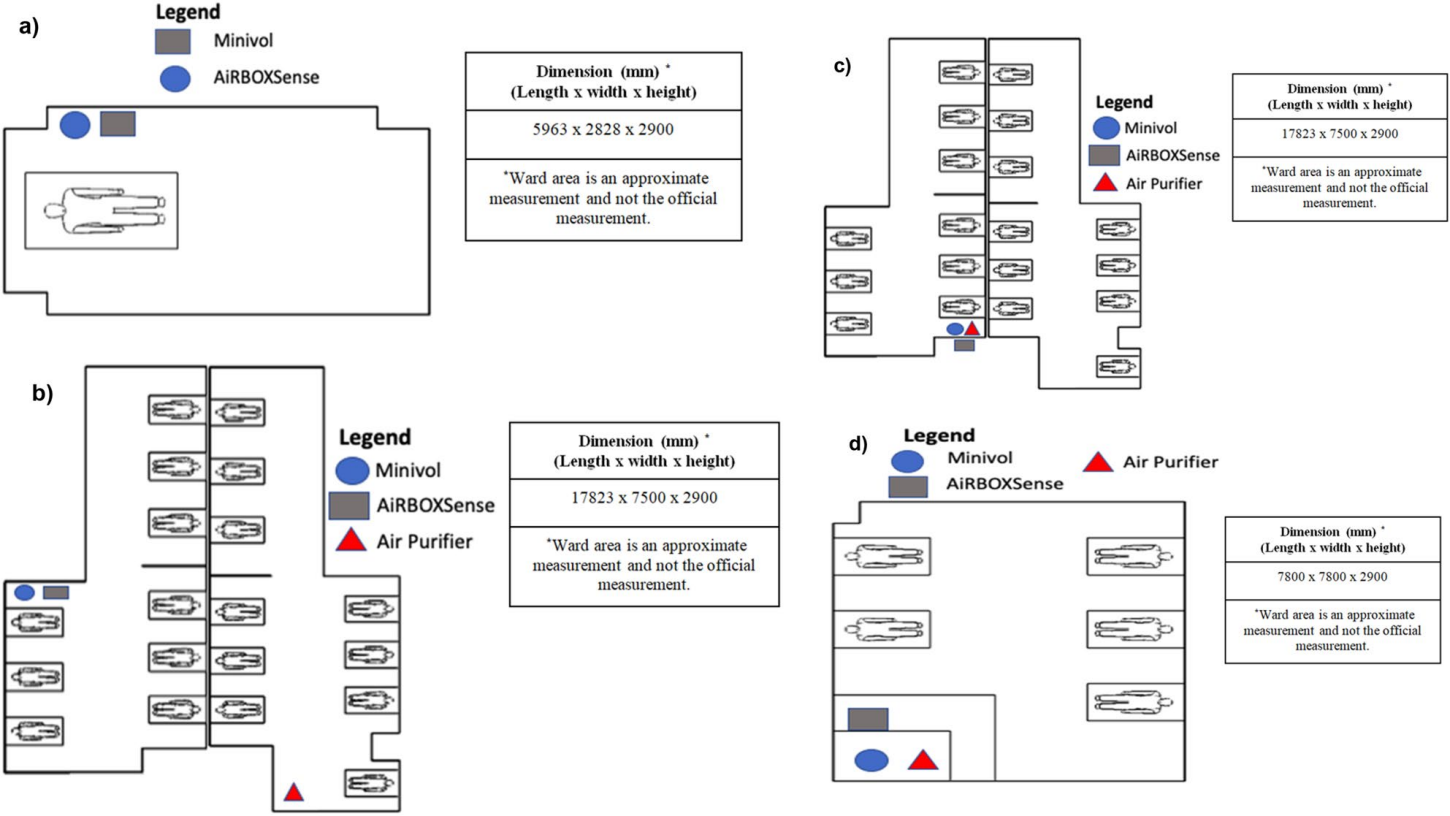
The layout and dimension of the wards with instrumentation deployment **a** single room A **b** general ward B, **c** general ward C and **d** general ward D. (Note: The beds in the figure does not represent the actual number of beds in the wards).

The layout and dimension of the wards are shown in Fig. 1. Each ward had different clusters of infected groups, as illustrated in Table 2 (in the main text) and Fig. 1. Each ward was occupied by one to eighteen COVID-19 patients. As a caveat, during the measurement in this study, hospital’s management staff has deployed three units of air purifiers at ward B, C, and D. During the air sampling measurement, air purifier (FANFIL AP510M, Aire-plus Technology, Singapore) was deployed at ~ 1 m distance in wards C and D, ~ 8 m in ward B, and no air purifier in single occupant room.

PM_2.5_ was sampled in a single-bed ward (31st March to 4th April 2020) and multiple bed wards (4th–29th April 2020) in a teaching hospital at Kuala Lumpur, respectively. Air sampling was conducted for 48 h during a 29 days sampling period using two types of instruments; an air quality sensor known as *AiRBOXSense* (AIRBOXSENSE V3.0, UKM Tech. Sdn Bhd, Malaysia)^12^^,^^26^ and a low volume sampler (LVS) (MINIVOL, AirMetrics, USA). Details of *AiRBOXSense* are described in^26^. Both instruments were operated side by side in wards occupied by SARS-CoV-2 positive patients. Instruments were treated using ultraviolet light for 20 min (UV) (UV-C 253.7 nm), further disinfected with 70% alcohol and calibrated before being translocated to the next wards. The same instruments were used to avoid variability during sampling.

*AiRBOXSense* was used to continuously measure PM_2.5_, while the LVS was used to determine the virus loading in PM_2.5_ trapped on filter paper (WHATMAN glass microfiber filters, Grade GF/F) with a tight specification of 0.6–0.8 μm particle retention and pure borosilicate glass structure, GF/F. A 5 L min^−1^ of air was drawn into the *AiRBOXSense*. While, the Minivol’s pump draws air at 5 L min^−1^ through a filter paper. The continuous concentration of PM_2.5_ was recorded and stored in secure digital card (SD card) in the *AiRBOXSense*. The data synchronously retrieved via THINGSPEAK (The MathWorks Inc, USA) cloud storage and analysed using MATLAB software (The MathWorks Inc, USA).

Each filter paper was collected after 48 h of sampling and stored in a sealed container and kept in − 80 °C laboratory freezer. The filter papers were extracted for viral load analysis using reverse transcription quantitative real time polymerase chain reaction (RT-qPCR) approach.

### Calibration of *AiRBOXsense*

*AiRBOXSense* was calibrated according to^26^ 1 day before each sampling. Calibration consists of setting a mathematical model describing the relationship between sensor data and reference instruments. The *AiRBOXSense* unit was calibrated in tandem with the GRIMM (as reference instrument) dust monitor model 1.108 (GRIMM Aerosol, Technik GmbH & Co. KG, Germany). The sensors measuring mass concentration were calibrated using GRIMM Aerosol, which was deployed at a clean area (laboratory) for lower concentration measurement and near to a car exhaust for high concentration measurement. The calibration equations are set by fitting a model during a calibration time interval when *AiRBOXSense* and GRIMM are co-located.

### Viral nucleic acid extraction

Prior to viral nucleic acid extraction, the membrane filter was processed according to^35^ with slight modifications. The membrane was first divided into four parts and immersed in 1 mL sterile RNase-free water in separate tubes. Each part of the membrane was vortexed for 2 min in 30 s-intervals to release viral particles attached to the membrane. The tubes were then centrifuged at 500 rpm for 1 min to remove debris, and the supernatants were transferred into new microcentrifuge tubes for viral nucleic acid extraction. This process was repeated twice to ensure all virus particles were resuspended into the water. Subsequently, viral nucleic acid extraction was performed using a Viral Nucleic Acid Extraction Kit II (Geneaid Biotech Ltd., Taiwan) according to the manufacturer’s protocol. The purified nucleic acid containing the samples was then kept at − 80 °C for further analysis.

### Reverse-transcription quantitative real-time polymerase chain reaction (RT-qPCR) analysis

The primers and probes used in the detection of SARS-CoV-2 were 2019-nCoV_N1, and 2019-nCoV_N2 combined primer/probe mixes purchased from Integrated DNA Technology (IDT). The information on primers and probes were included in Table 1. Human RNase P primer was not included as a control in this analysis because this study was not conducted using specimen from human. RT-qPCR was carried out using a THUNDERBIRD One-step RT-qPCR kit (Toyobo Co., Ltd., Japan) according to the manufacturer’s protocol. The annealing temperature of the primers was set at 55 °C, as suggested by Centres for Disease Control and Prevention or CDC (2020)^28^. Detection of SARS-CoV-2 using the RT-qPCR approach with a BIORAD iQ5 Real-Time PCR machine (BIORAD, USA) as described by CDC (2020) with slight modifications. A standard curve was also generated using 2019-nCoV Positive Control (nCoVPC) with a series of tenfold dilutions from 2 × 10^5^ to 2 copies/µL of the control template. The amplification efficiency and R^2^ value were recorded, and the standard curve was used to estimate the viral RNA of SARS-CoV-2 on the membrane.

**Table 1:** Primers and probes sequences.

Name of primers and probes	Description	Sequence (5′–3′)
2019-nCoV_N1-F	2019-nCoV_N1 Forward Primer	GAC CCC AAA ATC AGC GAA AT
2019-nCoV_N1-R	2019-nCoV_N1 Reverse Primer	TCT GGT TAC TGC CAG TTG AAT CTG
2019-nCoV_N1-P	2019-nCoV_N1 Probe	FAM-ACC CCG CAT TAC GTT TGG TGG ACC-BHQ1
2019-nCoV_N2-F	2019-nCoV_N2 Forward Primer	TTA CAA ACA TTG GCC GCA AA
2019-nCoV_N2-R	2019-nCoV_N2 Reverse Primer	GCG CGA CAT TCC GAA GAA
2019-nCoV_N2-P	2019-nCoV_N2 Probe	FAM-ACA ATT TGC CCC CAG CGC TTC AG-BHQ1

## Results and discussion

### Indoor PM_2.5_

**Figure 2:**
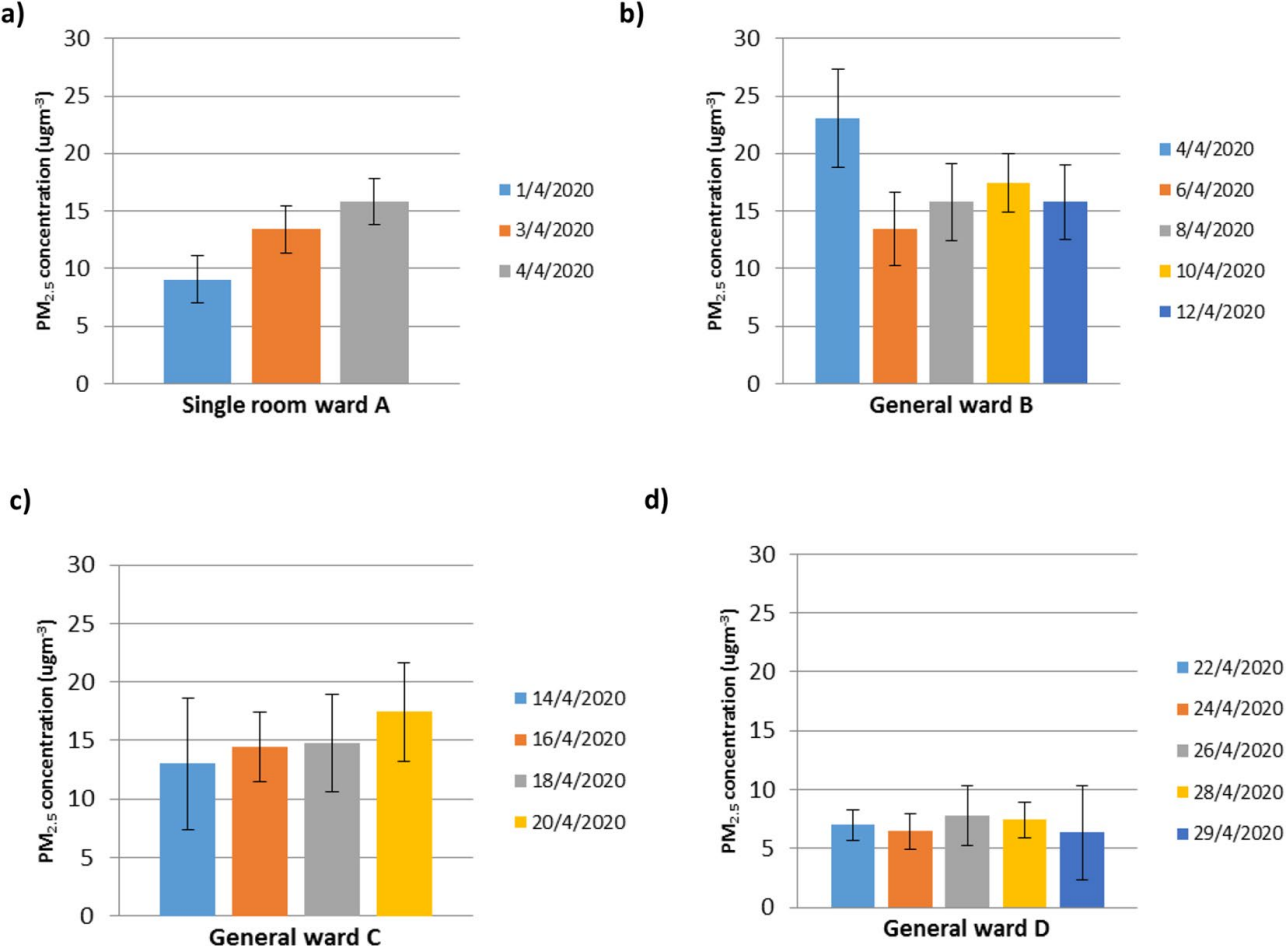
48 h average concentration PM_2.5_ at **a** single room ward A **b** general ward B, **c** general ward C and **d** general ward D.

All 48 h average of PM_2.5_ concentration measurements and samplings were taken in COVID-19 wards as illustrated in Fig. 2 and Table 2. The highest concentration of indoor PM_2.5_ was measured in general ward B (23.27 µg m^−3^) on the 4th April, while the lowest 48 h average concentration was measured in general ward D (6.23 µg m^−3^) on the 22nd April as shown in Fig. 2. The General ward B was occupied by a cluster of patients from the same institution and was observed to have the most activity among the patients. Higher PM_2.5_ concentrations can be contributed by physical activities such as movements of health workers and patients^21,27,29^. The PM_2.5_ concentrations measured in this study are slightly lower than reported in a European urban hospital^30^.

**Table 2:** Summary of the data collected from a teaching hospital at Kuala Lumpur.

Ward^a^	PM_2.5_ (µg m^−3^)^b^	SARS-CoV-2 RNA^c^	No. of occupied beds	Remarks
Single room Ward A	11.25 ± 2.05	Detected	1	Without air purifier
General Ward B	17.58 ± 4.27	Detected	18	Two air purifiers (LVS sampler located far from the air purifier)
General Ward C	14.66 ± 5.59	Not detected	17	One air purifier
General Ward D	7.57 ± 1.37	Not detected	8	One air purifier

^a^Selected wards that were sampled consisting of different patient clusters: Single room ward A, an executive ward that hosts only one COVID-19 patient; General ward B was occupied by an institutional cluster; General Ward C was occupied by patients arriving from overseas; and General Ward D was occupied by migrant workers.

^b^Average 48 hourly concentrations (with standard deviation) of PM_2.5_ measured in different wards.

^c^Detection of SARS-CoV-2 RNA on captured PM_2.5_ at different wards.

### Virus RNA analysis

SARS-CoV-2 RNA was isolated from filter membranes collected from the LVS. Only the N1 nucleocapsid gene was successfully detected in RT-qPCR in certain wards. According to the Emergency Use Authorization (EUA), detection of either the N1 or N2 gene is considered positive for the presence of SARS-CoV-2^30^. We detected positive results for SARS-CoV-2 genes in the single room Ward A (74 ± 117.1 copies μL^−1^) and General Ward B (10 ± 7.44 copies μL^−1^). The viral genomes extracted from the filter paper were of heterogenous mixture. This contributes to the high standard deviation in the virus copy number as heterogeneous nucleic acid template was used in RT-qPCR and the presence of SARS-CoV-2 genome was relatively low. Nonetheless, the cycle threshold (CT) value was < 40^30^, confirming the positive detection of SARS-CoV-2 in our samples (Table 2). Due to operational restriction imposed by the hospital, the sample size was limited and replication was not possible.

The uniqueness in the result is that viral RNA was still able to be detected in the single occupancy ward (Ward A). Ward A is a small enclosed room (22 m^2^) with a lavatory attached. The frequent use of the lavatory by the symptomatic patient is likely to result in the increase of viral shedding activity in the room. We suspect that virus-laden PM_2.5_ generated from the shedding activity circulated within the enclosed room despite low PM_2.5_ concentration (11.25 µg m^−3^), thus explaining the spike in the data. The degree of viral shedding (from the patients) due to symptoms such as coughing, sneezing, diarrhoea, etc. has been reported to influence the number of virus particles in the environment^1,5^. It is suggested that the increased virus particles (due to shedding) in a poorly ventilated environment might increase the virus-PM_2.5_ assemblage^9,19,31^. A study done by^5^ reported that they were not able to detect SARS-CoV-2 in all of their tested air samples. However, they highlighted that their short sampling time of 15 min–4 h might not represent total air volume in the ward and the presence of SARS-CoV-2 might have possibly been diluted during air exchanges in the ward. In contrast, viral RNA was able to be detected in this study when air sampling duration was extended.

SARS-CoV-2 RNA was also detected in General Ward B. General Ward B is a larger room (~ 100 m^2^) consisting of 18 occupied beds with two air purifying units installed at a distance of farther away from the LVS. The amount of SARS-CoV-2 collected in the particulate matter is significantly lower than from Ward A despite the higher number of patients and concentration of PM_2.5_ (17.58 µg m^−3^). Such a low viral load in the PM_2.5_ could be attributed to the minimal viral shredding despite the high particulate matter. These particulate matters suspended in the air could be derived from floor and surfaces^32,33^ as a result of the high occupants’ activities in ward B.

Virus-laden PM_2.5_ was not detected in Wards C and D despite having similar ward size. The number of patients in Ward C is similar to Ward B, whereas the number of patients in Ward D is half of that of Wards C. The patients in Ward C and Ward D were also diagnosed with mild symptoms. The non-detection of the virus in these wards may be due to very low virus shedding from the patients. Another possible factor to explain the absence of SARS-CoV-2 RNA in PM_2.5_ is that the LVS in Ward C (and also Ward D) was positioned adjacent to an air purifier. Although air-purifier’s effectiveness in removing PM_2.5_ remains unclear, air-filtration has been reported to reduce viral loading in air^9,32,33^.

Our results clearly indicated that SARS-CoV-2 RNA is present within sampling of the Ambient’s particles. Hence, it is crucial to determine whether these RNAs came from intact virus particles or are merely RNA from non-infectious virus particles. The detection of SARS-CoV-2 viral RNA on surfaces was previously reported on a cruise ship, the Diamond Prince, even after 17 days after the evacuation of passengers^34^. In addition, the CDC pointed out that the infectivity of the detected particles was still uncertain. A study carried out in a CDC facility showed that SARS-CoV-2 could remain infectious up to 72 h on various types of surfaces^24^. Thus, it is suggested that infectious virus be determined by culturing of virus residing on the PM_2.5_ onto appropriate cell culture. However, our study could not show a direct link between the concentration of PM_2.5_ and SARS-CoV-2. We did find that PM_2.5_ generated from human activities in healthcare facilities can influence the presence of SARS-CoV-2 RNA in indoor environments. Furthermore, the degree of viral shedding from symptomatic patients may also influence the presence of SARS-CoV-2 RNA on PM_2.5_. Therefore, we recommend that all possible precautions against airborne transmission in indoor environments should be taken seriously.
